# Sleep Quality and Quality of Life in COPD Patients with and without Suspected Obstructive Sleep Apnea

**DOI:** 10.1155/2014/508372

**Published:** 2014-01-22

**Authors:** Mohammad Ali Zohal, Zohreh Yazdi, Amir Mohammad Kazemifar, Parisa Mahjoob, Masomeh Ziaeeha

**Affiliations:** ^1^Department of Internal Medicine, Metabolic Disease Research Center, Qazvin University of Medical Sciences, Qazvin 3419759811, Iran; ^2^Department of Internal Medicine, Qazvin University of Medical Sciences, Qazvin 3419759811, Iran; ^3^Department of Health, Qazvin University of Medical Sciences, Qazvin 3419759811, Iran

## Abstract

Present study was designed to obtain association between sleep apnea with sleep quality and quality of life in COPD patients. This cross-sectional descriptive study was conducted on 139 patients with COPD in a chest clinic of a university hospital. All patients were evaluated by pulmonary function test for determination of severity of their disease. Also, Berlin questionnaire, Epworth sleepiness scale, Pittsburgh Sleep Quality Index, and St. George Respiratory questionnaires (SGRQ) were employed for assessment of patients. Analysis of data showed that quality of sleep was significantly correlated with quality of life (*P* < 0.001). About half of the patients were at high risk for sleep apnea. The patients were divided into two groups according to the result of Berlin questionnaire. Significant differences were found between the groups for total score and each of three subscores of SGRQ suggesting worse quality of life in overlap syndrome (*P* < 0.001). Also, patients with overlap syndrome had worse quality of sleep compared to patients without it (8.1 ± 1.7 versus 6.2 ± 2.3; *P* < 0.001). Stepwise multiple regression analysis showed that severity of COPD, coexisting obstructive sleep apnea, and sleep quality accounted for the SGRQ significantly (*r* ^2^ (coefficient of determination) = 0.08, 0.21, and 0.18, resp.). It is recommended that patient with COPD be evaluated for sleep apnea and sleep disorders during routine examinations and followups.

## 1. Introduction

Both chronic obstructive pulmonary disease and obstructive sleep apnea are common [1]. It is estimated that 5–15% of adult population suffers from COPD [2]. Also, obstructive sleep apnea (OSA) is prevalent in adults with 4% of men and 2% of women [3]. Then simultaneous presentation of them, that is, overlap syndrome, is not surprising [4].

Both diseases may affect sleep quality and quality of life. Chronic obstructive pulmonary disease may produce subjective and objective changes in sleep. The patients have difficulties in initiation and continuance of sleep and may complain from daytime sleepiness. Moreover, nocturnal drops in O_2_ saturation caused by COPD eventually may lead to pulmonary hypertension, cardiac arrhythmia, and night time arousal [5, 6].

The nocturnal drop in O_2_ saturation in patients with overlap syndrome may lead to the more severe daytime sleepiness, shorter sleep time, lower sleep efficiency and higher arousal index are expected. However, it is essential to note that hypoxia is one of the causes of these symptoms in the patients. Recent studies have demonstrated that quality of sleep is under influence of obstructive apnea rather than severity of airways obstruction in overlap syndrome [7–9]. Also some authors have reported that sleep disorders have reduced quality of sleep in patients with COPD [9, 10]. A study performed on patients with COPD has shown that 70% of the patients have poor sleep quality. In this study, quality of life was correlated with sleep quality, FEV_1_ after bronchodilator administration, and severity of dyspnea, while sleep quality was the most considerable variable [11].

Another study has evaluated quality of life and sleep quality in COPD using St. George questionnaire and a general questionnaire for assessment of quality of life and Pittsburg questionnaire for evaluation of sleep quality [9]. It showed that sleep quality is an independent prognostic factor for quality of life, though it found no relationship between severity of COPD and quality of life or sleep quality. Furthermore, results of a recent study have implied that patients with COPD have shorter sleep duration, more prolonged initiation of sleep, more frequent arousal, and lower nocturnal O_2_ saturation, compared to control [12]. Evaluation of quality of life in overlap syndrome has been subject of a recent study [10]. St. George questionnaire was used for evaluation of patients with overlap syndrome and COPD. All of the patients had night snoring without significant daytime sleepiness. Also patients with overlap syndrome had lower quality of life, compared to patients with COPD. Study of Johnson and his coworkers in Sweden has also showed that 51% of patients with COPD have sleep disorder, while 31% of control group had the problem. They used questionnaire as well as polysomnography for evaluation of their patients [13].

There are insufficient studies in the literature to reach definite conclusion about the matter. Hence, present study was conducted to evaluate quality of life and sleep quality in patients with COPD and overlap syndrome.

## 2. Materials and Methods

Present cross-sectional study was performed on patients with COPD who consequently referred to a chest clinic in a teaching university hospital in Qazvin city, Iran between January 2011 and August 2011. Severity of their diseases was determined according to criteria defined by American thoracic society, after their consent for participation into the study (FEV1 ≥ 80% and FEV1/FVC < 70%, mild; FEV1/FVC < 70% and 50 ≤ FEV1 < 80, moderate; and FEV1 < 50% and FEV1/FVC < 70%, severe).

Demographic characteristics of the patients including their age, gender, occupation, level of education, cigarette smoking, drug history, and coexisting disorders were recorded.

Pittsburg questionnaire was used for assessment of sleep quality. It assesses patient's view about his/her sleep quality during the last 4 weeks with 9 questions. The answers are scored in 7 divisions from 0 to 3. The total score would be 0 to 21. If the score is higher than 5, it will indicate inadequate sleep quality [14].

Berlin questionnaire was utilized for evaluation of sleep apnea in the patients. It contains 10 questions about risk factors of sleep apnea such as snoring, day time sleepiness and fatigue, obesity, and hypertension. If the individual scores positive in at least 2 of the 3 categories, he/she will be at high risk of sleep apnea [15].

Epworth sleepiness scale (ESS) questionnaire was employed for analysis of daytime sleepiness in the patients. It comprises 8 questions that depict situations in which the individual may become sleepy reluctantly. Each question takes 0 to 3 scores. If the patient's score is higher than 10, he/she will have more than usual daytime sleepiness [16].

St. George questionnaire was used for assessment of quality of life of the patients. It contains 76 items about symptoms, impact, and activity of the disease, which provide total score according to index of effect of any subscore, from 0 to 100 for individual subscores and totally. Higher score means lower quality of life in those subscores. It has been used in many diseases included lung disease such as asthma and COPD. Content of its questions is simple and easily understood by patients. It has accepted statistical validity.

Differences in continuous variables between groups (different severities of COPD and patients with and without OSA) were assessed by student's *t*-test and ANOVA. Also, Chi-square test was used to determine the association between categorical variables (sex and number of patients suspected to OSA) with different severities of COPD. Logistic regression analysis was used to investigate the relationship between quality of life as dependent variables and other independent variables (severity of COPD, coexisting obstructive sleep apnea, and sleep quality). A *P* value of less than 0.05 was considered statistically significant.

## 3. Results

In the present study, 139 patients (89 males and 50 females) with COPD participated. Their age was 66.4 ± 10.8 years. Their BMI was 25.9 ± 4.9 kg/m^2^. The value of FEV_1_ in the patients was 1.39 ± 1.1 lit. Main characteristics of the studied patients divided by severity of their disease are demonstrated in Table 1.

Analysis of Pittsburg questionnaires showed that 104 patients (74.8%) had poor sleep quality, while only 35 (25.2%) had appropriate sleep quality. Moreover, 71 patients (51.1%) were at risk of sleep apnea. Sleep quality and quality of life in patients with or without sleep apnea are shown in Table 2. Relationship between quality of sleep and suspected sleep apnea is also demonstrated in Table 3. As the table shows, the proportion of patients with suspected sleep apnea that reported poor quality of sleep (44.6%) was significantly higher than patients without sleep apnea (30.2%) (*P* < 0.001).

Finally, stepwise multiple regression analysis showed that severity of COPD, coexisting obstructive sleep apnea, and sleep quality accounted for the SGRQ significantly (*r* ^2^ (coefficient of determination) = 0.08, 0.21, and 0.18, resp.).

## 4. Discussion

Quality of life is a key feature in evaluation of severity of diseases and their response to the treatments. It is regarded as a tool for evaluation of the patients in numerous studies [17–19]. Results of the present study confirmed that quality of life worsens with progression of the disease in patients with COPD. Furthermore, coexisting obstructive sleep apnea exacerbates the effect. Progressive and restraining natures of the disease affect capabilities of the patients and their quality of life in COPD [9, 10, 17–19].

In the current study, about half of the patients had coexisting suspected obstructive sleep apnea. It is far higher than estimated levels for normal population [20].

In the present study, numerous patients had poor sleep quality, which exacerbated with increase in severity of the disease and coexisting obstructive sleep apnea. It is consistent with earlier related studies. Muniz and his colleagues have performed a study on relationship between quality of life and sleep quality in patients with COPD. They have found that 70% of the patients had poor sleep quality [11]. Scharf and his coworkers have also reported that mean score of the patients with COPD is 11 ± 5.4 using Pittsburg questionnaire, which means poor sleep quality. In this study, 77% had sleep disorders, while sleep quality was independent prognostic factor for quality of life [9]. A variety of factors such as nocturnal cough, dyspnea, drugs for instance theophylline, and age-related changes contribute to this [19, 21].

The present study showed that quality of life and sleep quality are poorer in patients with overlap syndrome, compared to patients with COPD alone. It is consistent with a previous related study which had used polysomnography for evaluation of sleep apnea [10]. It has showed that patients with overlap syndrome are at higher risk of respiratory failure and pulmonary hypertension, when compared to patients with obstructive sleep apnea alone. In these patients, nocturnal hypoxemia was more severe too, particularly in patients with COPD.

Evaluation of our patients for quality of life has revealed that the worst scores were obtained from section of physical activity. It is in agreement with preceding related studies [10, 17]. Limitations in physical and social activities resulting from COPD are indisputable. Presence of intolerable respiratory symptoms and their emotional consequences also have impact on quality of life.

There were limitations in the present study. Polysomnography was not performed for the patients due to technical shortage, so Berlin's questionnaire was used instead. Our study was designed as cross-sectional type. Perhaps cohort study would attain more conclusive results for relationship between sleep quality and quality of life.

In summary, results of the present study confirmed that obstructive sleep apnea, poor sleep quality, and lower quality of life are highly prevalent in COPD patients. Also, presence of OSA in COPD patients is correlated with lower scores of sleep quality and worse quality of life.

According to the results, it is recommended that patients with COPD be evaluated for clinical symptoms and signs of obstructive sleep apnea such as obesity, increased neck circumference, hypertension, and snoring. Screening and diagnostic tools should be used, if indicated.

## Figures and Tables

**Table 1:** Major characteristics of the studied patients divided by severity of their disease.

	Mild *n* = 39	Moderate *n* = 58	Severe *n* = 42
Age*	59.4 ± 12.7	63.6 ± 9.4	68.3 ± 8.3
Sex**			
Male	25 (17.9%)	37 (26.6%)	27 (19.4%)
Female	14 (10.1%)	21 (15.1%)	15 (10.8%)
Body mass index (Kg/m^2^)*	27.3 ± 2.6	25.3 ± 4.1	24.4 ± 3.2
FEV_1_ (liter)*	1.98 ± 0.7	1.43 ± 1.1	1.21 ± 1.3
ESS scores*	9.1 ± 1.4	12.8 ± 0.9	14.7 ± 2.1
PSQI scores*	7.5 ± 1.2	6.9 ± 0.9	9.1 ± 1.5
Quality of life (total)*	43.5 ± 12.5	49.1 ± 9.4	53.9 ± 14.2
Symptoms*	47.6 ± 9.3	53.4 ± 8.3	58.9 ± 13.2
Activity*	57.3 ± 8.9	63.7 ± 12.6	60.8 ± 11.3
Impact*	38.4 ± 9.3	41.3 ± 7.9	43.2 ± 8.7
Number of patients suspected to OSA**	15 (21.1%)	25 (35.2%)	31 (43.6%)

*Data are presented as mean ± standard deviation (SD). **Data are presented as number (percentage).

**Table 2:** Sleep quality, quality of life, and sleepiness in studied patients with or without obstructive sleep apnea (OSA).

	Patients with OSA *n* = 71	Patients without OSA *n* = 68	*P* value
PSQI scores*	8.1 ± 1.7	6.2 ± 2.3	<0.001
Quality of life*	60.6 ± 10.4	40.2 ± 11.8	<0.001
Symptoms*	61.1 ± 12.9	52.8 ± 15	<0.001
Activity*	72.5 ± 13.9	49.9 ± 16.04	0.002
Impact*	53.6 ± 11.9	30.5 ± 11.8	<0.001
ESS scores*	10.9 ± 8.9	8.9 ± 2.6	0.005

*Data are presented as mean ± standard deviation (SD).

**Table 3:** Relationship between quality of sleep and obstructive sleep apnea (OSA) in studied patients.

	Patients with OSA *n* = 71	Patients without OSA *n* = 68	*P* value
Good sleep quality	9 (6.4%)	26 (18.7%)	<0.001
Poor sleep quality	62 (44.6%)	42 (30.2%)	

*Data are presented as number (percent).
